# Editorial: Bioinformatics and big data driving the discovery of clinical biomarkers for solid tumors and blood malignancies

**DOI:** 10.3389/fgene.2026.1999998

**Published:** 2026-10-20

**Authors:** Maoshan Chen, Jing Zhang, Xiaotao Jiang

**Affiliations:** 1 Department of Blood Transfusion, Laboratory Medicine Centre, The Second Affiliated Hospital, Army Medical University, Chongqing, China; 2 Hematopoietic Acute Radiation Syndrome Medical and Pharmaceutical Basic Research Innovation Center, Ministry of Education of the People’s Republic of China, Chongqing, China; 3 Microbiome Research Centre, School of Clinical Medicine, Faculty of Medicine and Health, University of New South Wales (UNSW), Sydney, NSW, Australia; 4 University of New South Wales, Kensington, NSW, Australia

**Keywords:** clinical biomarker, hematologic malignancy, liquid biopsy, machine learning, multi-omics, single-cell, solid tumor

## Introduction

An association between a molecular feature and a clinical outcome is a hypothesis, not a biomarker. Turning high-throughput profiling into a clinically actionable marker means integrating heterogeneous data layers and validating outside the discovery cohort. The 11 original research articles in this Research Topic cover breast, ovarian, renal, lung, pancreatic and skin cancers alongside diffuse large B-cell lymphoma (DLBCL) and chronic myeloid leukemia (CML). Methodologically, they range from bulk machine-learning risk models to single-cell, imaging and comorbidity-based clinical prediction (Figure 1).

**FIGURE 1:**
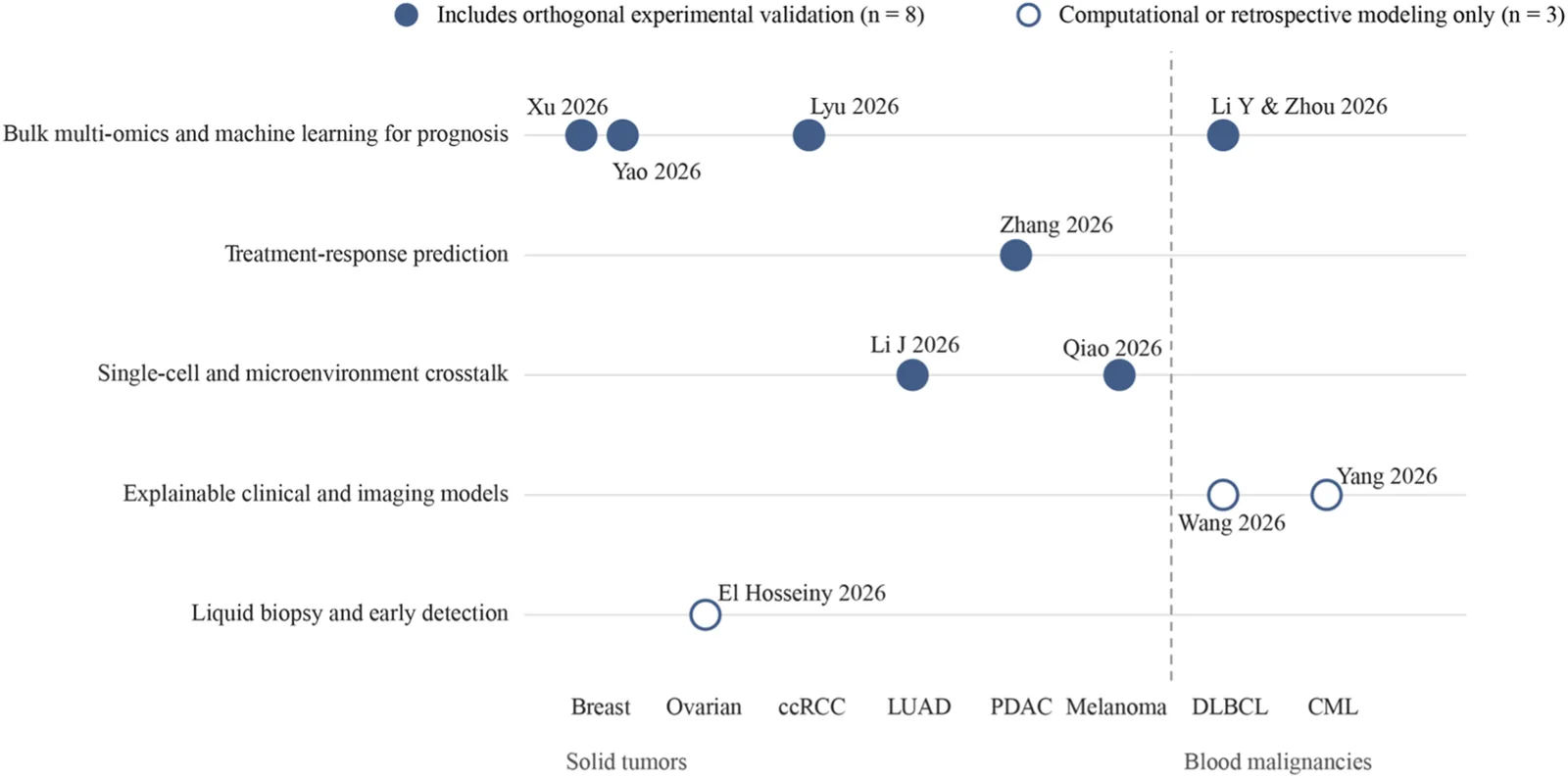
The 11 studies contributing to this Research Topic, arranged by disease context and analytical theme. Studies are grouped by theme (rows) and by disease (columns), with solid tumors separated from hematologic malignancies by the dashed line. Filled circles indicate studies that include orthogonal experimental validation (RT-qPCR, immunohistochemistry, immunofluorescence or functional assays); open circles indicate studies based on computational or retrospective clinical modeling alone.

The prognostic models here rest on bulk transcriptomes and machine learning. Xu et al. combined weighted gene co-expression network analysis, protein–protein interaction networks and LASSO-Cox regression to define a six-gene NETosis- and inflammation-related model in breast cancer (*ZMYND10*, *IL12B*, *CXCL13*, *TFF1*, *LTB*, *SPIB*). The risk score remained independent of age and other clinical features; five of the six genes were confirmed by RT-qPCR, *LTB* excluded for limited tissue. Yao et al. derived and validated a seven-gene metastasis-associated signature (*IGJ*, *CXCL14*, *PTGER3*, *RTN1*, *EGOT*, *TLR10*, *PANX2*) in TCGA-BRCA, AURORA US, SCAN-B and GEO cohorts; low-risk tumors were immune-hot, with activated CD8^+^ T cells, whereas high-risk tumors showed T-cell exclusion and kinase and metabolic enrichment. *RTN1* and *TLR10* are proposed as candidate drivers awaiting functional confirmation. In clear-cell renal cell carcinoma, Lyu et al. built a nucleotide-metabolism signature from single-cell and bulk transcriptomics, reportedly outperforming 122 published models; *IFI30* was validated *in vitro* and *in vivo*.

Two studies move from prognosis to treatment response. Li Q et al. derived a pretreatment 15-gene score predicting non-pathological complete response to neoadjuvant chemotherapy in breast cancer (AUC = 0.809 and 0.876 in two external cohorts) and traced the signal to a pyruvate–*CCND1*–*YAP1*–*HIF1A*/*ARNT*–MET axis: serum pyruvate was elevated in non-pCR tumors, and capmatinib, a MET inhibitor, attenuated pyruvate-driven phenotypes. In pancreatic ductal adenocarcinoma, Zhang et al. combined lactylation- and programmed-cell-death-related genes to identify *HMGA1* and *KIF2C* as core prognostic drivers, supporting the model with drug-sensitivity and clinical specimens. Both nominate an actionable pathway alongside their risk score.

For blood cancers, three studies cover molecular prognosis, imaging prediction and clinical risk scoring, complementing transcriptome-derived prognostic sets in multiple myeloma and acute lymphoblastic leukemia (Hou et al., 2026; Tang et al., 2025). Li and Zhou reported that *SYTL4* was upregulated in DLBCL and independently associated with adverse outcome (multivariate HR = 25.5, 95% CI 1.8–367.3; *P* = 0.017; *n* = 48); the width of that interval reflects the small tissue-microarray subgroup. *SYTL4*-high tumors were enriched for extracellular matrix remodeling, whereas mitochondrial translation and oxidative phosphorylation were negatively enriched. Wang et al. trained deep learning models on 18F-FDG PET/CT PyRadiomics features to predict cancer-related cognitive impairment in DLBCL without CNS involvement; the single-center design and modest sample (*n* = 112) make external validation a prerequisite. Yang et al. compared three comorbidity indices (CCI, ACE-27, CIRS-G) in 559 patients on flumatinib for chronic-phase CML. CIRS-G contributed the largest incremental value for 12-month major molecular response (ΔAUC = 0.078), and SHAP analysis exposed a non-linear threshold (CIRS-G ≥ 8) above which predicted response declined, though the authors frame these findings as exploratory.

Liquid biopsy contributes one candidate panel. El Hosseiny et al. identified a circulating three-miRNA panel (*hsa-miR-29b-3p*, *hsa-miR-19b-3p*, *hsa-miR-30e-5p*) for early-stage ovarian cancer from public miRNA-seq data, with a random-forest classifier reaching 91.7% accuracy (AUC = 0.991) and pathway analysis implicating TP53 and VEGFA signaling. Platelets and extracellular vesicles carry tumour-derived material, including in cholangiocarcinoma, and raise the same analytical-versus-diagnostic validity question (Chen et al., 2022; Green et al., 2025; Zhang and Chen, 2026). Such retrospective numbers are hypothesis-generating at best, and confirmation in screening and symptomatic populations is still missing; this was also the only contribution to compare signatures across cancer types.

Two single-cell studies resolve microenvironment crosstalk. Qiao et al. profiled 70,760 cells from 11 melanoma samples, resolved nine subpopulations, and used CellChat to show that prognostically favorable subpopulations engaged MIF–CD74/CD44/CXCR4 and MHC-I signaling, with CD8^+^ T cells as the predominant receivers; a 15-gene signature reached a mean C-index of 0.675, and high *EIF5A* expression was associated with poor outcome. Li J et al. combined single-cell transcriptomics with Mendelian randomization in lung adenocarcinoma, identifying seven prognostic genes related to endoplasmic reticulum stress and butyrate metabolism (*VDAC1*, *TXNRD1*, *GDF15*, *TRIB3*, *LPL*, *KCNQ1*, *PKP2*), validated by RT-qPCR, with macrophages central to progression. Both studies turn up candidates that bulk profiling would average away.

## Perspectives

The most conspicuous gap is prospective validation. Most studies here rest on public repositories or single-institution retrospective cohorts, and until multicenter prospective Research Topic with harmonized preprocessing becomes routine, none of these signatures is ready for a clinical guideline.

Spatial multi-omics is the natural next step, connecting single-cell inference to tissue architecture (Yu et al., 2022). Signatures that account for regional heterogeneity, immune-excluded versus inflamed niches, for example, should generalize better than those from homogenized bulk tissue, and coupling spatial context with liquid-biopsy dynamics could enable non-invasive tracking of spatial evolution. Spatial profiling is absent from this Research Topic, though it was an explicit aim of the call.

Multimodal models that encode genomics, imaging, clinical text and longitudinal outcomes in one framework are appealing, but their bias, calibration and failure modes need explicit evaluation before clinical deployment, a discipline now being formalized beyond oncology (Ibisanmi et al., 2026). Reporting standards also need tightening: TRIPOD + AI (Collins et al., 2024) for prediction models and CLEAR (Kocak et al., 2023) for radiomics should be routine rather than optional, and explainability through SHAP attribution, attention weights or pathway mapping belongs in the main text, not the supplement.

Taken together, these papers suggest that useful cancer biomarkers will rarely be single molecules. They combine multi-omics features with circulating or imaging signals, microenvironment context and defined clinical endpoints, and the computation holding them together has to be transparent. Eight of the 11 pair a computational hypothesis with orthogonal validation, which makes them useful templates for the path from a bioinformatics discovery to a clinical tool.
